# CD93 is expressed on chronic myeloid leukemia stem cells and identifies a quiescent population which persists after tyrosine kinase inhibitor therapy

**DOI:** 10.1038/s41375-019-0684-5

**Published:** 2020-01-02

**Authors:** Ross Kinstrie, Gillian A. Horne, Heather Morrison, David Irvine, Chinmay Munje, Eduardo Gómez Castañeda, Hothri A. Moka, Karen Dunn, Jennifer E. Cassels, Narissa Parry, Cassie J. Clarke, Mary T. Scott, Richard E. Clark, Tessa L. Holyoake, Helen Wheadon, Mhairi Copland

**Affiliations:** 1 Paul O’Gorman Leukaemia Research Centre, Institute of Cancer Sciences, University of Glasgow, Glasgow, UK; 2 Molecular and Clinical Cancer Medicine, University of Liverpool, Liverpool, UK

**Keywords:** Cancer stem cells, Chronic myeloid leukaemia

## Abstract

The introduction of BCR-ABL tyrosine kinase inhibitors has revolutionized the treatment of chronic myeloid leukemia (CML). A major clinical aim remains the identification and elimination of low-level disease persistence, termed “minimal residual disease”. The phenomenon of disease persistence suggests that despite targeted therapeutic approaches, BCR-ABL-independent mechanisms exist which sustain the survival of leukemic stem cells (LSCs). Although other markers of a primitive CML LSC population have been identified in the preclinical setting, only CD26 appears to offer clinical utility. Here we demonstrate consistent and selective expression of CD93 on a lin^−^CD34^+^CD38^−^CD90^+^ CML LSC population and show in vitro and in vivo data to suggest increased stem cell characteristics, as well as robust engraftment in patient-derived xenograft models in comparison with a CD93^−^ CML stem/progenitor cell population, which fails to engraft. Through bulk and single-cell analyses of selected stem cell and cell survival-specific genes, we confirmed the quiescent character and demonstrate their persistence in a population of CML patient samples who demonstrate molecular relapse on TKI withdrawal. Taken together, our results identify that CD93 is consistently and selectively expressed on a lin^−^CD34^+^CD38^−^CD90^+^ CML LSC population with stem cell characteristics and may be an important indicator in determining poor TKI responders.

## Introduction

Chronic myeloid leukemia (CML) originates from a constitutively active tyrosine kinase, BCR-ABL. In CML, not all leukemia stem cells (LSCs) are eradicated by tyrosine kinase inhibitors (TKIs) in vitro and a population of lin^−^CD34^+^ CML progenitors have the ability to remain quiescent and engraft NSG mice [1–4]. Furthermore, cells of a similar phenotype have been identified in the bone marrow (BM) of imatinib-treated CML patients in complete cytogenetic response [5]. These findings verify CML LSCs are not absolutely dependent on BCR-ABL activity for their survival, and may determine disease persistence, highlighting those patients who are at high risk of molecular recurrence on TKI withdrawal [6, 7]. Although many labs have performed extensive analyses to identify potential surface markers of primitive cell populations in the preclinical setting, including CD26 [8–10], and IL1-RAP [11, 12], these markers show variability and have, therefore, not yet been translated into routine clinical practice. However, CD26 is promising, with recent data suggesting a correlation between CD26 expression and treatment response, as well as a Lin^−^CD34^+^CD38^−/low^CD45RA^−^cKIT^−^CD26^+^ population being identified as a potential therapeutic target at a single-cell level [13]; the diagnostic potential of CD26 is currently being evaluated within clinical trials [14, 15].

We present for the first time, evidence for the role of CD93 as a primitive marker with functional relevance in chronic phase (CP)-CML LSCs. A variety of functions for CD93 have been described, including leukocyte migration, and cell adhesion, and it has been identified on a number of cell types, including cells of a myeloid origin, stem cells, endothelial cells, and platelets [16, 17]. Despite this, its purpose and mechanisms in myeloid malignancy have yet to be fully elucidated. It has, however, been shown to offer potential as a biomarker for an AML LSC population in MLL-rearranged AML [18]. Here, we demonstrate consistent and selective expression of CD93 on a lin^−^CD34^+^CD38^−^CD90^+^ CP-CML LSC population and show robust engraftment of this population in patient-derived xenograft (PDX) models in comparison with CD93^−^ CML stem/progenitor cells, which fail to engraft, confirming its relevance in CP-CML.

## Methods

### Human samples

Informed consent was obtained in accordance with the Declaration of Helsinki and with approval from Greater Glasgow and Clyde NHS Trust Ethics Committee. BM samples from trial entry of the DESTINY clinical trial (NCT01804985) [19] were utilized to assess cell populations in patients with/without molecular recurrence on TKI discontinuation. Sample details are listed in Table S1. CD34^+^ cells were purified and cryopreserved as previously described [20]. A minimum of three biological replicates were performed for each experiment in the first instance with more biological replicates included if patient heterogeneity was observed.

Prior to FACS sorting, CD34^+^ cells were thawed over 20 min in DAMP solution and incubated overnight in serum free medium with high growth factors (SFM + HGF) to maximize recovery post thaw, as previously described [2]. Following overnight incubation, CD34^+^ cells were cultured in a ‘physiological’ growth factor cocktail (1 in 100 dilution, SFM + HGF).

### Drugs and reagents

Imatinib, dasatinib, and nilotinib (all LC laboratories) were made into stock solutions of 10 mM in DMSO. Dilutions to working concentrations were made with media.

### Flow cytometry and cell sorting

Cells were stained using the following antibody cocktail (all BD Biosciences apart from CD93-PE from eBioscience); lineage cocktail-FITC [CD3 (MφP9), CD14 (3G8), CD16 (NCAM16.2), CD19 (SJ25C1), CD20 (SK7), CD56 (L27)], CD34-PerCP (8G12), CD38-V450 (HIT2), CD45RA-APC H7 (HI100), CD90-PE Cy7 (5E10), CD123-APC (7G3), and CD93-PE (R3). Immunophenotypic analysis and cell sorting of normal and CML samples was performed following antibody staining on a FACSCanto or FACSAria (BD Biosciences). FACS data were analyzed with FACS Diva software (Becton Dickinson) or FlowJo (TreeStar).

### In vitro colony-forming cell (CFC), replating and long-term culture-initiating cell (LTC-IC) assays

2000 FACS-sorted cells from stem and progenitor subpopulations were plated in duplicate in Methocult optimum (H4034, Stem Cell Technologies). Following incubation at 37° for 10–12 days, colonies were counted. For replating, 50 individual primary colonies per sample were picked and re-suspended in 200 μl Methocult in 96 well plates, incubated at 37° for 10–12 days before positive wells counted. Primary CP-CML samples were sorted for Lin^−^CD34^+^CD93^−/+^ cells and cultured overnight. Cells were washed and inoculated into pre-prepared long-term cultures comprising a stromal feeder layer (1:1 mix of irradiated (80 Gy) SL/SL and M210B4 murine fibroblasts) in long-term myeloid culture medium (MyeloCult supplemented with hydrocortisone; Stem Cell Technologies) [21]. Cultures were maintained for 5 weeks with 50% media changes performed weekly. Each well was harvested, counted, and seeded into Methocult to perform CFC assays.

### Murine xenotransplantation assay

Experiments were performed in accordance with the local ethical review panel, the UK Home Office Animals Scientific Procedures Act, 1986, and UK Coordinating Committee on Cancer Research and National Cancer Research Institute guidelines. Animals were kept in regulated facilities, monitored daily, and all experiments complied with UK Home Office guidelines. Mice were genotyped by Transnetyx. NOD.Cg-*Prkdc*^*scid*^*IL2rg*^*tm1Wjl*^/SzJ (NSG) mice were purchased from Charles River Laboratories, UK. Sample size was dictated by cell number from primary samples. No randomization or blinding was used during murine xenotransplantation assays.

The NSG mouse model was used to assay human LSCs with in vivo engraftment capacity (SCID-repopulating cells or SRC). CP-CML lin^−^CD34^+^CD93^+^ or lin^−^CD34^+^CD93^−^ cells were isolated by FACS (1 × 10^6^ cells/mouse), washed and transplanted via tail vein injection into sub-lethally irradiated (200 cGy) 8–12-week-old NSG mice. Mice were euthanized after 16 weeks and BM obtained. To assess human cell engraftment, cells were labeled with anti-human CD45 (H130), CD33 (P67.6), and CD19 (SJ25C1) antibodies (all BD Biosciences) prior to analysis by flow cytometry. Human CD45^+^CD33^+^ cells were isolated by FACS and analyzed by FISH for the *BCR-ABL* gene rearrangement. For in vivo TKI treatments, NSG mice were transplanted with 1 × 10^6^ FACS-sorted CD34^+^ cells as above, and left for 12 weeks to allow engraftment. Mice were then treated with 50 mg/kg nilotinib by oral gavage once daily for 28 days before euthanizing and BM extracted. Human cells were analyzed by flow cytometry following labeling with anti-human antibodies against CD45 (H130), CD33 (P67.6), CD34 (8G12), CD38 (HIT2) all BD Biosciences, and CD93 (R3) (eBiosciences).

### Microarray and bioinformatic analysis

Cell populations were FACS-sorted into: Hematopoietic stem cell (HSC)/LSC (Lin^−^CD34^+^CD38^−^CD45RA^−^CD90^+^), multipotent progenitor (MPP; Lin^−^CD34^+^CD38^−^CD45RA^−^CD90^−^), common myeloid progenitor (CMP; Lin^−^CD34^+^CD38^+^CD45RA^−^CD123^+^), granulocyte-macrophage progenitor (GMP; Lin^−^CD34^+^CD38^+^CD45RA^+^CD123^+^), and megakaryocyte-erythroid progenitor (MEP; Lin^−^CD34^+^CD38^+^CD45RA^−^CD123^−^). RNA extracted from stem/progenitor cell subpopulations was analyzed with Affymetrix Human Gene 1.0 ST arrays (GEO accession number GSE47927 [22]) by Polyomics, University of Glasgow. Using R/Bioconductor, data were RMA normalized and subsequently analyzed using a modified LIMMA protocol [23]. Differential expression was identified by LIMMA using *q* < 0.05.

### Cytogenetics and fluorescence in situ hybridization (FISH) analysis

FISH was performed with the LS1 *BCR-ABL* Dual Color FISH probe (Abbott Diagnostics) according to the manufacturer’s instructions. Results are presented as percent of positive interphases as calculated in a minimum of 100 cells.

### RT-PCR

RNA was extracted using the Qiagen RNEasy Minikit as per the manufacturer’s instructions and reverse transcribed using the high-capacity cDNA synthesis kit (Applied Biosystems). Primers were designed using NCBI software (Table S2). Quantitative RT-PCR was performed on a Taqman 7900 instrument (Applied Biosystems) and using Fluidigm technology. Gene expression was determined relative to four housekeeping genes and expressed as 2^−ΔCt^ or compared with an untreated calibrator (2^−ΔΔCt^) [24]. Multiplex PCR was performed as per [25].

### Single-cell analysis of transcription

RNA was extracted from lin^−^CD34^+^, lin^−^CD34^+^CD38^−^CD90^+^CD93^−^, and lin^−^CD34^+^CD38^−^CD90^+^CD93^+^ populations, reverse transcribed, and 14 cycles of gene-specific amplification performed using the Applied Biosystem pre-amplification kit with relevant primer sets. Following amplification, 2 uL of the resultant product was used for multiplex PCR reaction, as described [25]. The rest of the resultant products were loaded in triplicate onto pre-primed 96 × 96 Fluidigm microfluidic dynamic arrays and analyzed according to the manufacturer’s instructions. To assess single-cell gene expression, Fluidigm C1™ was used. Analysis was performed using R 3.3.3 under macOS 10.13.2. Data processing and normalization were performed independently for each chip. Only those genes with detectable expression in at least 10 CD93^+^ and 10 CD93^−^ cells within the same chip were analyzed. All the expression values were normalized using the –ΔΔCt method [24].

### Statistical analysis

Statistics were calculated using R-3.4.3 and Prism 6 software (GraphPad Software, Inc). Data are presented as the mean ± SD. Statistical significance was determined via Student’s *t* test (*p* < 0.05 was considered significant). Single-cell statistical analysis was performed using Kolmogorov–Smirnov test and corrected for multiple comparisons using Benjamini–Hochberg method [23, 26]. The correlation between the –ΔCts and the frequency of detectable expression was performed using Pearson’s r correlation coefficient.

## Results

### CML stem cells are more proliferative than normal HSCs

**Fig. 1:**
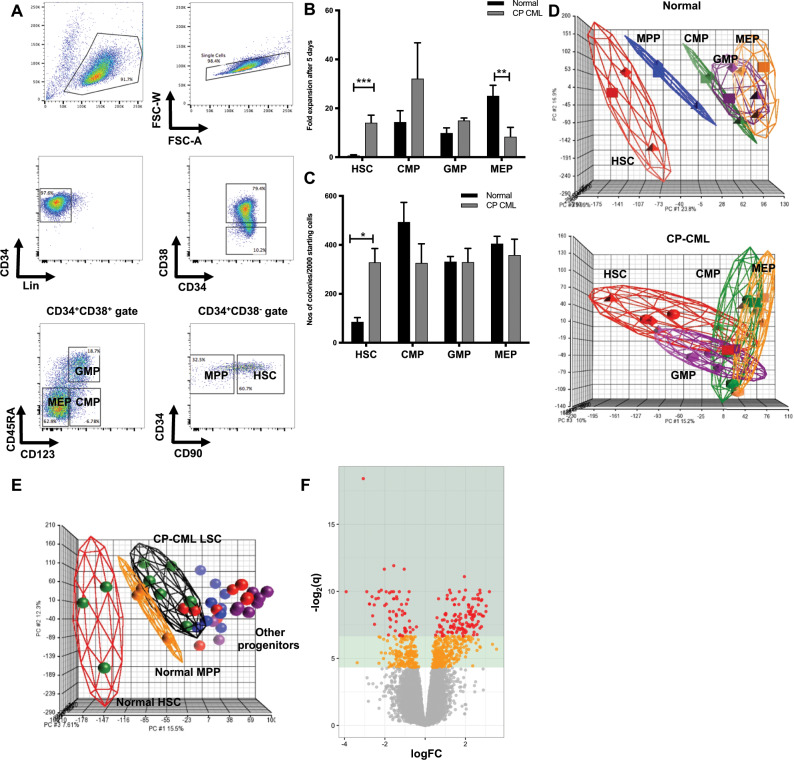
CML LSCs are more proliferative than normal HSCs. **a** A representative sorting strategy of CP-CML samples is depicted. **b** Fold expansion after 5 days (mean ± standard deviation) are shown (**p < 0.01; ***p < 0.001). **c** Colony counts are represented (mean ± standard deviation) per 2000 starting cells to allow comparison between groups (*p < 0.05). **d** Principal component analysis (PCA) of the gene expression profiles from GSE47927 was used to identify global differences between normal and CP-CML. An individual point within the axes represents each microarray and each subpopulation grouping by a different color. Separate PCAs of normal and CP-CML subpopulations. **e** PCA of normal and CP samples overlapped. **f** A volcano plot from the same dataset showing the deregulation of genes in a comparison of Lin^−^CD34^+^CD38^−^CD90^+^ cells from six CP-CML samples compared with three normal samples (GSE47927). Areas of significance are indicated by a dark green (equivalent to *q* ≤ 0.01) or light green (equivalent to *q* ≤ 0.05) background. Genes reaching significance are colored red (*q* ≤ 0.01) or orange (*q* ≤ 0.05); non-significant genes are gray.

To determine transcriptomic and functional differences between CML LSCs and normal HSCs, normal (*n* = 3) and CP-CML (*n* = 6) samples were FACS-sorted into HSC/LSC, CMP, GMP, and MEP subpopulations (Fig. 1a). Within the HSC/LSC compartment, CML LSCs demonstrated significantly increased proliferation (14-fold expansion, *p* < 0.001) compared with normal HSCs (no expansion) after 5 days in vitro culture in physiological growth factors (Fig. 1b). There was variable expansion in both normal and CML CMP and GMP subpopulations, with no significant difference between CML and normal. Expansion of CML MEP was significantly reduced compared with normal. Equivalent numbers of CML LSCs produced approximately fourfold more colonies in CFC assays than normal HSCs (329 ± 46 versus 86 ± 17 per 2000 cells, respectively; *p* < 0.05) (Fig. 1c). There was no significant difference between normal and CML CMP, GMP, and MEP subpopulations in the number of colonies produced. FISH demonstrated that >90% of CML LSCs from all patient samples were *BCR-ABL* positive.

### CML LSCs have a less primitive gene expression profile than normal HSCs

Next, we characterized the gene expression profiles of the different CML and normal subpopulations (GEO accession number GSE47927). Data were RMA normalized and analyzed using a modified LIMMA/Bioconductor protocol. The gene expression profile of CML LSCs was determined to be more akin to normal primitive progenitor cells, and not normal HSCs, as represented through Principal Component Analyses (PCA) where, when plotted together, LSCs from patients with CP-CML (*n* = 6) have a global gene expression signature that appears more mature than normal HSCs (*n* = 3) and is positioned overlapping both normal and CML progenitor subpopulations (Fig. 1d, e). Although not surprising that CP-CML LSCs display a myeloid-based transcriptome compared with normal HSCs, it confirms the ability of mature progenitor populations to behave as ‘stem cells’ in the CML model. Within this dataset (GSE47927), 1217 genes were deregulated between CP-CML LSCs and normal HSCs (Fig. 1f).

**Fig. 2:**
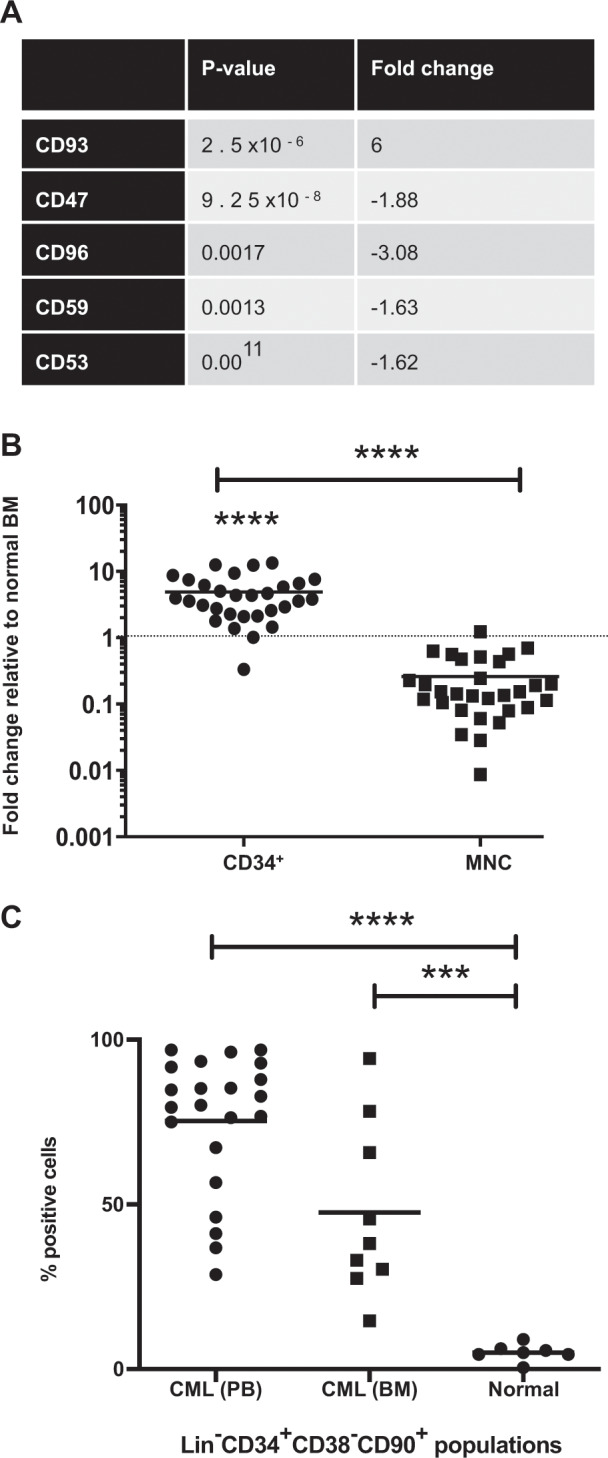
CD93 expression can isolate a functional stem cell population in CML. **a** Analysis of the microarray, GSE47927, demonstrated a significant fold change between CP-CML LSCs and normal HSCs in genes for cell surface proteins. The table represents the most statistically significant cell surface marker genes identified. **b** RNA from 30 BM and PB CP-CML samples was utilized to validate the gene expression of *CD93*. MNC and CD34^+^-selected (*n* = 30) samples were compared with normal BM (*n* = 5). **c** CD93 protein expression was assessed by flow cytometry in lin^−^CD34^+^CD38^−^CD90^+^ populations between CML (peripheral blood [*n* = 22] and BM [*n* = 9]) and normal BM (*n* = 7). A statistically significant increase in percentage of CD93^+^ cells in CML was determined using an unpaired Student’s *t* test (*p* < 0.0001). MNC; mononuclear cells, PB; peripheral blood, BM; bone marrow.

We utilized this gene expression dataset to evaluate the expression of cell surface markers. Using *z* scores to normalize the gene expression for each sample, we plotted heatmaps using R/Bioconductor. This demonstrated the variable expression of cell surface markers between CP-CML LSCs and normal HSCs (Fig. S1). Cell surface markers expressed were significantly altered between the CML LSC and normal HSC populations (statistically significant cell surface markers shown in Fig. 2a). Of note, *CD93* demonstrated a sixfold increased expression in CP-CML LSCs compared with normal HSCs (*p* = 2.5 × 10^−6^). Gene expression of *CD26* and *IL1-RAP* were not significantly different between the Lin^−^CD34^+^CD38^−^CD45RA^−^CD90^+^ LSC and HSC populations, perhaps due to the immature phenotype of the cell type being analyzed.

### CD93 is upregulated in CP-CML cells

Upregulation of CD93 gene expression was confirmed by quantitative RT-PCR in both CD34^+^-selected peripheral blood (PB) and BM (*n* = 30), compared with normal BM samples (*n* = 5) (*p* < 0.0001; Fig. 2b). There was a significant decrease in *CD93* expression with maturation from CD34^+^ to mononuclear cells (MNC) (*p* < 0.0001) in CML, indicating that high expression of *CD93* may be a specific marker of primitive LSC in CML.

Next, we assessed CD93 protein expression on the LSC population isolated from CP-CML PB samples (*n* = 22), and CP-CML BM (*n* = 9) compared with normal samples (*n* = 7) using FACS analysis and confirmed the significant upregulation of CD93 protein on CML LSCs compared with normal HSCs (*p* < 0.0001), where CD93 expression was absent/very low (Fig. 2c). Thus, we hypothesized that the identification of CD93 may define a functional LSC population.

### CD93^+^, but not CD93^−^, CP-CML cells have stem cell characteristics in vitro and in vivo

**Fig. 3:**
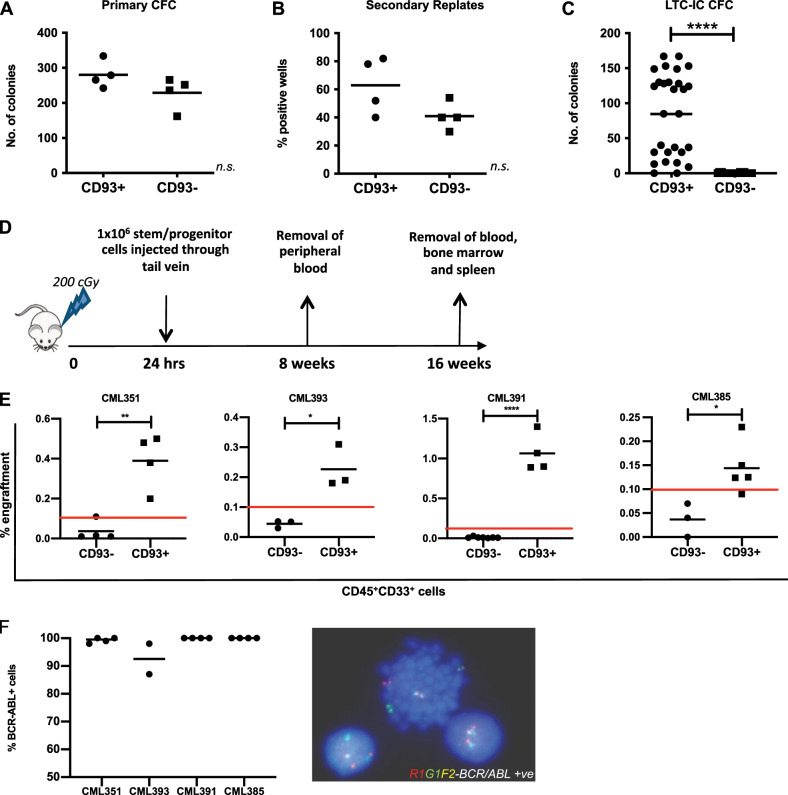
CD93^+^, but not CD93^−^, CP-CML cells have stem cell characteristics in vitro and in vivo. **a** CP-CML samples (*n* = 4) were sorted according to Fig. S2 and plated in duplicate into Methocult for 10–12 days prior to colony counts. The graph indicates the mean number of colonies for each CP-CML sample, with mean and standard deviation for each population shown. **b** 50 primary colonies per sample were replated in Methocult in 96 well plates and incubated at 37°, 5% CO_2_ for 12 days before positive wells were counted. The graph indicates the percentage of positive wells per population (*n* = 4 CP-CML samples), with mean and standard deviation shown. **c** The graph indicates the number of colonies from each experimental arm (*n* = 3 CP-CML samples), with mean and standard deviation shown. **d** Representation of in vivo experimental model. CP-CML Lin^−^CD34^+^CD93^+^ or Lin^−^CD34^+^CD93^−^ cells were isolated by FACS sorting (1 × 10^6^ cells/mouse), washed and transplanted via tail vein injection into sub-lethally irradiated (2 Gy) 8–12-week-old NSG mice. PB was sampled at 8 weeks to assess for CD45^+^33^+^ expression. All mice were euthanized after 16 weeks and marrow contents of femurs were obtained. Cells were labeled with anti-human CD45, CD33, and CD19 antibodies prior to analysis by flow cytometry. This allowed for analysis of lineages within the engrafted samples. **e** Human myeloid cell engraftment was characterized by the percentage of CD45^+^CD33^+^ cells. **f** Human CD45^+^ cells were isolated by FACS sorting and analyzed by FISH for the *BCR-ABL* gene rearrangement. Percentage of *BCR-ABL* positive cells, as determined from analysis of a minimum of 100 cells, was assessed for each murine experiment.

To assess the LSC potential of CD93^+^ and CD93^−^ stem/progenitor cells in vitro, CFC replating and LTC-IC assays were performed on primary CP-CML samples sorted into Lin^−^CD34^+^CD38^-^CD90^+^CD93^+^ (LSC-CD93^+^) and Lin^−^CD34^+^CD38^−^CD90^+^CD93^−^ (LSC-CD93^−^) populations (Fig. S2). There was little difference in the ability of LSC-CD93^+^ and LSC-CD93^−^ to form CFCs in primary assays (Fig. 3a). This perhaps reflects the ability of committed progenitors to form colonies. Therefore, to assess self-renewal, 50 primary colonies were picked, dispersed, and transferred to secondary replating assays and counted after 12 days in culture. The counts demonstrated a trend toward an increase in percentage of positive wells in the LSC-CD93^+^ population compared with LSC-CD93^−^ (*p* = 0.09; Fig. 3b), suggesting remaining LSC-CD93^+^ cells from primary CFC plating have enhanced colony formation in secondary CFC assays, likely through a more primitive phenotype, compared with their LSC-CD93^−^ counterparts.

To interrogate this further, the LTC-IC assay was utilized. Results demonstrated a statistically significant increase in colonies within the Lin^−^CD34^+^CD93^+^ population with no, or few, colonies within the Lin^−^CD34^+^CD93^−^ population (*p* < 0.0001; Fig. 3c). This validated our previous finding that CD93^+^-selected cells have a more primitive phenotype, with increased self-renewal capacity in vitro compared with CD93^−^-selected CML cells. To determine whether this was due to differences in *BCR-ABL* status, the two cell populations, Lin^−^CD34^+^CD93^+^ and Lin^−^CD34^+^CD93^-^ were interrogated by FISH for *BCR-ABL* status. Analyzed cells from both fractions were shown to be 100% *BCR-ABL* positive (data not shown).

To validate whether Lin^−^CD34^+^CD93^+^ truly represented a more primitive CP-CML LSC fraction than Lin^−^CD34^+^CD93^−^, xenotransplantation experiments using NSG recipient mice were conducted. Prior to sorting, CP-CML samples (*n* = 5) were determined to be >98% *BCR-ABL* positive by FISH (Fig. S3). In experiments similar to those described by Herrmann et al. for CD26 [10], Lin^−^CD34^+^CD93^+^ or Lin^−^CD34^+^CD93^−^ cells were transplanted via tail vein injection into sub-lethally irradiated (2 Gy) 8–12-week-old NSG mice (Fig. 3d). At 8 weeks post transplant, PB was removed from the mice and analyzed for engraftment using CD45 and CD33 expression by flow cytometry (*n* = 3). There was no significant difference in CD45^+^CD33^+^ expression between the Lin^−^CD34^+^CD93^+^ and Lin^−^CD34^+^CD93^−^ populations at this early time point (Fig. S4). After 16 weeks, there was a statistically significant increase in engraftment of human CD45^+^CD33^+^ cells within the BM of mice transplanted with Lin^−^CD34^+^CD93^+^ cells from four independent CP-CML samples (CML351 *p* = 0.015; CML393 *p* < 0.05; CML391 *p* < 0.0001; CML385 *p* = 0.0119) (Fig. 3e). Engraftment levels were consistent with other CP-CML murine NSG models [10, 27, 28]. Human CD45^+^CD33^+^ cells were isolated post engraftment from NSG murine BM and analyzed by FISH for the *BCR-ABL* gene rearrangement. These samples were *BCR-ABL* positive (Fig. 3f) demonstrating that the CD93^+^-selected population has an increased self-renewal capacity and has stem cell-like characteristics.

Within a further CP-CML sample, there was no difference in engraftment (Fig. S5). However, engrafted cells from the CD93^−^ subpopulation were found to be of both lymphoid and myeloid lineages and *BCR-ABL* negative, with pre-engraftment FISH demonstrating a small, but biologically significant percentage of *BCR-ABL* negative cells (CML395; Fig. S3).

Taken together, these in vitro and in vivo results suggest that the CD93^+^ population represents a more primitive self-renewing population of CP-CML LSCs than CD93^−^ cells and is a more immature precursor population within the leukemia stem and progenitor cell hierarchy.

**Fig. 4:**
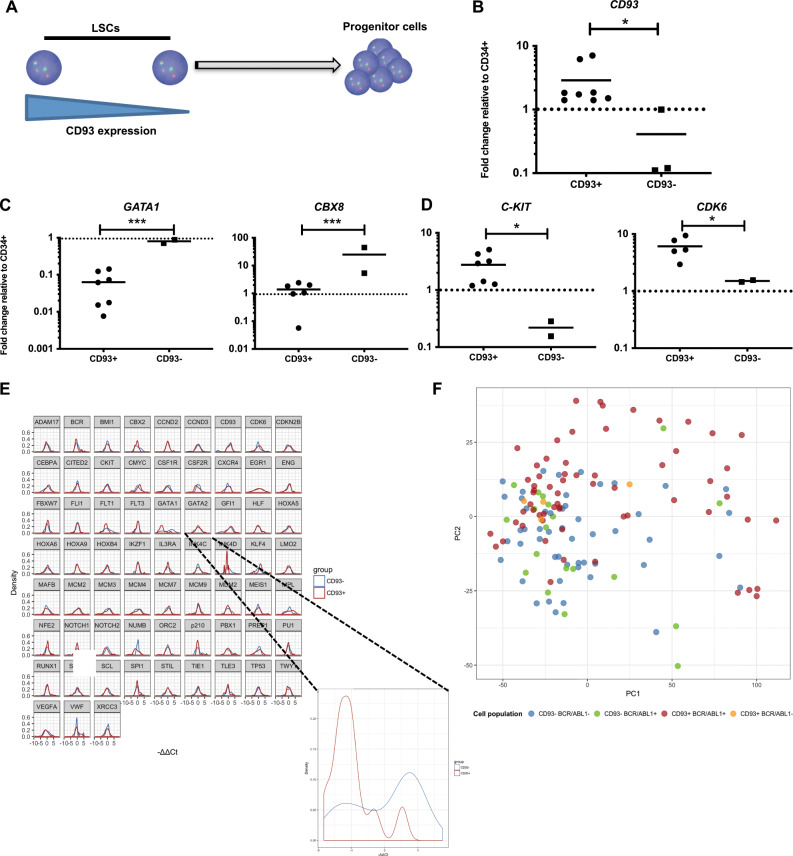
Gene expression profiling of bulk CD93-selected CP-CML LSCs confirms their quiescent character. **a** Schematic diagram of CD93 expression within a stem cell population. **b** *CD93* gene expression as determined in the lin^−^CD34^+^CD38^−^CD90^+^CD93^+^ and lin^−^CD34^+^CD38^−^CD90^+^CD93^−^ population (*p* = 0.0068). Results are presented as fold change compared with the CD34^+^ bulk population using the ΔΔCt method. **c** Key genes involved in lineage restriction were overexpressed in the lin^−^CD34^+^CD38^−^CD90^+^CD93^−^ population, namely *GATA1* and *CBX8*. **d** Key genes involved in stem cell maintenance were upregulated in the lin^−^CD34^+^CD38^−^CD90^+^CD93^+^ population. **e** Normalized differential gene expression of the single cells using the ΔΔCt method. The control sample is the mean of the CD93^−^ on each chip. *GATA1* was significantly downregulated in the CD93^+^ population (*n* = 2 CP-CML samples, *q* = 0.03). **f** Principal component analysis (PCA) of the four different populations defined by CD93 surface presence and *BCR-ABL1* expression. Each individual dot represents a single cell. It can be observed that the CD93^+^ *BCR-ABL1*^*+*^ cells (in red) are more heterogeneous than the other populations and that they form a slightly different cluster.

As CD93^+^-selected populations have functional properties of CML LSC, with evidence of increased engraftment potential, we hypothesized that those cells with less CD93 expression would have a more mature and less lineage-restricted gene expression profile compared with LSC-CD93^+^ cells. It was expected that as CD93 positivity represents a spectrum of expression rather than a binary system, that gene expression would also display this. Any statistical results, therefore, would be extremely significant (Fig. 4a). Using Fluidigm targeted gene analysis of 48 cell survival and self-renewal genes, *CD93* was demonstrated to be upregulated within the FACS-sorted LSC-CD93^+^ population compared with LSC-CD93^−^ population (*p* = 0.0068) (Fig. 4b). A number of genes involved in lineage restriction and cell cycle were statistically different in the LSC-CD93^−^ population, including *GATA1* (*p* = 0.0007), *CBX8* (*p* = 0.0002), and *CYCLIND2* (*p* < 0.01) (Fig. 4 c). Moreover, the LSC-CD93^+^ population overexpressed a number of genes associated with HSC regulation, including *C-KIT* (*p* = 0.0014) and *CDK6 (p* = 0.05) (Fig. 4d).

We next utilized single-cell gene expression using Fluidigm C1 technology to corroborate these findings. CP-CML cells were sorted into (1) lin^−^CD34^+^, (2) LSC-CD93^−,^ and (3) LSC-CD93^+^ populations. Single cells were sorted into each population across two individual CP-CML samples (*n* = 150 LSC-CD93^+^, *n* = 150 LSC-CD93^−^ single cells. The frequency of expression [29] for each gene was determined. A total of 20 genes had a higher frequency within the CD93^+^, and 53 genes had a higher frequency within the CD93^−^-selected single-cell populations (Fig. S6). Next, a threshold of 10 events for population per chip was set for analyzing individual genes. Using a Kolmogorov–Smirnov test and correcting for false discovery rate with *p* < 0.05 [23], *GATA1* expression was significantly lower in the CD93^+^ single cells (Fig. 4e; Table 1), but no other significant changes were identified.

**Table 1:** Single-cell gene expression profiling identifies that CD93^+^-selected single cells have a stem cell signature.

Gene	*p* value	*q* value	Gene	*p* value	*q* value
*ADAM17*	0.273	0.650	*INK4D*	0.310	0.668
*BCR*	0.266	0.650	*KLF4*	0.018	0.336
*BMI1*	0.456	0.777	*LMO2*	0.951	0.992
*CBX2*	0.759	0.963	*MAFB*	0.723	0.955
*CCND2*	0.992	0.992	*MCM2*	0.264	0.650
*CCND3*	0.188	0.650	*MCM3*	0.459	0.777
*CD93*	0.900	0.992	*MCM4*	0.281	0.650
*CDK6*	0.267	0.650	*MCM7*	0.314	0.668
*CDKN2B*	0.268	0.650	*MCM9*	0.640	0.903
*CEBPA*	0.194	0.650	*MDM2*	0.657	0.903
*CITED2*	0.604	0.886	*MEIS1*	0.603	0.886
*C-KIT*	0.503	0.829	*MPL*	0.033	0.366
*CMYC*	0.011	0.336	*NFE2*	0.122	0.650
*CSF1R*	0.100	0.650	*NOTCH1*	0.447	0.777
*CSF2R*	0.040	0.366	*NOTCH2*	0.068	0.496
*CXCR4*	0.991	0.992	*NUMB*	0.020	0.336
*EGR1*	0.963	0.992	*ORC2*	0.914	0.992
*ENG*	0.880	0.992	*p210*	0.278	0.650
*FBXW7*	0.161	0.650	*PBX1*	0.561	0.886
*FLI1*	0.905	0.992	*PREP1*	0.590	0.886
*FLT1*	0.044	0.366	*PU1*	0.262	0.650
*FLT3*	0.942	0.992	*RUNX1*	0.286	0.650
*GATA1*	**0.0005**	**0.031**	*SCA1*	0.420	0.770
*GATA2*	0.394	0.764	*SCL*	0.646	0.903
*GFI1*	0.029	0.366	*SPI1*	0.140	0.650
*HLF*	0.970	0.992	*STIL*	0.983	0.992
*HOXA5*	0.709	0.955	*TIE1*	0.150	0.650
*HOXA6*	0.212	0.650	*TLE3*	0.352	0.726
*HOXA9*	0.749	0.963	*TP53*	0.394	0.764
*HOXB4*	0.817	0.992	*TWY1*	0.882	0.992
*IKZF1*	0.418	0.770	*VEGFA*	0.209	0.650
*IL3RA*	0.593	0.886	*VWF*	0.808	0.992
*INK4C*	0.207	0.650	*XRCC3*	0.247	0.650

Using a Kolmogorov–Smirnov test and correcting for false discovery rate with *p* < 0.05, *GATA1* expression was statistically significant (based on the *q* value) between CD93^−^ and CD93^+^ populations

The bold highlighted text is the only statistically significant result in Table 1

Furthermore, hierarchical gene clustering could not clearly discriminate between CD93^+^ and CD93^−^ populations at a single-cell level, and therefore highlighted the heterogeneity within the two populations by single-cell analysis (Fig. S7). Furthermore, PCA of four different populations defined by CD93 and *BCR-ABL1* expression demonstrated that the CD93^+^*BCR-ABL1*^*+*^ cells were more heterogeneous than the other populations (Fig. 4f). There was an increasing trend towards CD93^+^ cells being *BCR-ABL1*^+^ by multiplex PCR. The changes within frequency and gene expression are subtle within a single-cell approach and small changes or trends may be biologically important.

Overall, these data suggest that CD93^+^ selection confers cell immaturity and self-renewal capability, with the majority of this population retaining *BCR-ABL* expression.

### TKIs downregulate CD93, but do not eliminate CD93^+^ LSC even after prolonged in vivo therapy

**Fig. 5:**
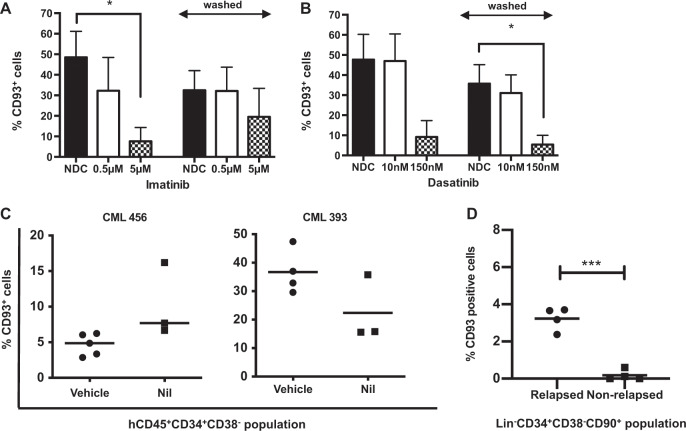
TKIs downregulate CD93, but do not eliminate CD93^+^ LSC even after prolonged in vivo therapy. CP-CML cells (*n* = 3) were thawed and sorted into an LSC population before culturing in SFM plus physiological growth factors with and without Imatinib (**a**) or Dasatinib (**b**). Following culture for 24 h, half of the samples were analyzed by flow cytometry to assess CD93 expression. The rest were thoroughly washed to completely remove TKI and re-cultured in SFM supplemented with physiological growth factors for a further 24 h prior to flow cytometry analysis. **c** A nilotinib-treated PDX model was used to determine CD93+ cells in the hCD45+CD34+CD38^−^ population by flow cytometry. This demonstrates persistence of CD93 expression despite TKI treatment in two patient samples. **d** CD93 expression as determined by flow cytometry in eight patients with prolonged TKI treatment. Within patients who relapsed post TKI discontinuation, a small lin^−^CD34^+^CD38^−^CD90^+^CD93^+^ population could be identified, compared with patients who did not demonstrate a molecular relapse.

We next sought to determine if CD93 expression would alter following TKI exposure. Lin^−^CD34^+^ CP-CML samples (*n* = 3) were cultured in the presence of imatinib or dasatinib. Baseline CD93 expression was analyzed by flow cytometry. After 24 h, cells were washed of TKI and cultured for a further 24 h before flow cytometric expression of CD93 was analyzed. Results suggested that TKIs reduce but do not eliminate CD93 expression (Fig. 5a, b). Next, CD93 expression was assessed in a Nilotinib-treated PDX model from two different patient samples. CD93 expression was assessed in the hCD45^+^CD34^+^CD38^−^ population. Again, TKI treatment did not eliminate CD93 expression (Fig. 5c). It was noted, however, that there was an interpatient variability in expression when using the PDX model. This led us to question if CD93 expression represents a self-renewing population in CP-CML cells, and if it is not eliminated by TKI, could it be detected in patients who have been treated by TKI and subsequently relapse following TKI withdrawal.

BM samples of patients in MR4 (BCR-ABL1:ABL1 ratio of <0.01%), taken at entry into the DESTINY clinical trial (NCT01804985) [19], who either remained in MMR or better for at least 3 years (*n* = 4) or developed molecular recurrence (*n* = 4) after TKI discontinuation were thawed and cultured as previously described. Samples were sorted into LSC-CD93^−^ and LSC-CD93^+^ populations. A significant increase in CD93 expressing LSCs were identified in patients with molecular recurrence compared with those in sustained MMR or better (Fig. 5d; *p* = 0.006); the majority of these cells were BCR-ABL1^+^ by FISH (Fig. S8A, B), suggesting a persisting LSC population.

## Discussion

We have presented functional and genomic data demonstrating that CD93 expression may be utilized to identify CML cells with stem cell-like capability. Further to this, we have identified that CD93 expression persists despite prolonged TKI exposure in those patients with molecular recurrence upon TKI discontinuation.

We initially identified the Lin^−^CD34^+^CD38^−^CD45RA^−^CD90^+^ CP-CML stem/progenitor subpopulation as having greater proliferative and colony-forming capacity than normal HSCs. CD93 had increased expression in CML LSCs compared with normal HSCs, and in vitro and in vivo analysis confirmed the stem cell-like capacity of the CP-CML CD93^+^ population. Initial in vitro analysis of clonal progenitor assays demonstrated minimal differences in colony-forming capacity between lin^−^CD34^+^CD38^−^CD90^+^CD93^+^ and lin^−^CD34^+^CD38^−^CD90^+^CD93^−^ populations. This is unsurprising as the ability to form a multilineage colony focuses on the ability to differentiate, with only limited involvement of self-renewal [30]. LTC-IC assays were utilized to determine quantitative assessment of LSC function [30, 31], as the most stringent in vitro surrogate measure of the functional activity of HSCs/LSCs [31]. Our results demonstrated a statistically significant increase in colonies within the lin^−^CD34^+^CD93^+^ experimental arm (*p* = 0.0001), suggesting that CD93^+^-selected cells have increased in vitro stem cell-like capacity. Because in vitro measurements of self-renewal do not reflect the complex multidirectional interactions seen within the BM, we utilized an in vivo NSG engraftment model to determine if the CD93^+^-selected population could be truly representative of an enhanced self-renewing population. Within four independent CP-CML samples, there was a statistically significant increase in engraftment of CD93^+^ compared with CD93^−^-selected populations. All levels of engraftment were within the previously published range for a CP-CML in vivo model [10, 27, 28]. This verified our previous findings that the CD93^+^-selected population has functional properties of LSCs, with the evidence of increased engraftment potential. This is an intriguing finding, as an LSC immunophenotype was not used for the LTC-IC assay, nor the in vivo component due to low cell numbers, suggesting that CD93 is discriminating between a functional LSC (i.e., those with self-renewing capability) and more mature cells within the stem cell compartment. Further to this, CD93 expression was not eliminated by TKI and persisted in patients with prolonged TKI exposure (>3 years) who developed molecular recurrence upon TKI withdrawal.

Many groups have attempted to identify novel cell surface markers of the CML LSC. Included in these are CD25, IL1-RAP, and CD26, identified within a CD34^+^CD38^−/low^ CML stem/progenitor cell population [9, 32, 33]. IL1-RAP and CD26 are arguably the most well established, and have been shown to separate *BCR-ABL* positive and negative cell populations, as well as being investigated for their potential for targeting therapeutically [10, 11, 34]. CD26 has shown promise within clinical translation, with data indicating CML LSCs specifically express CD26, with its function disrupting interactions within the stem cell niche [10], and its expression being correlated with both leukocyte count and treatment response [8]. Recent studies indicate the most TKI-insensitive cells can be captured within a Lin^−^CD34^+^CD38^−/low^CD45RA^−^cKIT^−^CD26^+^ population, suggesting this fraction of cells as a potential therapeutic target [13]. However, a recent publication has demonstrated that although vildagliptin, a dipeptidyl-peptidase IV (CD26) inhibitor, reduced LSC mobilization in an in vitro co-culture model, there was no significant effects in a NSG (PDX) model when combined with TKI therapy, perhaps highlighting the complexity of CD26 within a multidirectional niche system [35]. The initial identification of CD26 and IL1-RAP was based on a more mature cell population, namely CD34^+^CD38^−/low^, and not the very primitive Lin^−^CD34^+^CD38^−^CD45RA^−^CD90^+^ evaluated here. Therefore, differences in CD93 expression would be missed in these previous studies as its preferential expression on LSCs lies within a significantly more primitive population. This was highlighted in a recent study by Warfvinge et al., where CD93 expression was shown within the MNC fraction of both normal and CML samples [13]. Our data similarly demonstrated that within a MNC population there is limited expression of CD93 in both normal and CML. However, our findings of increased CD93 expression and its importance is based on its presence on the LSC population, immunophenotypically defined as Lin^−^CD34^+^CD38^−^CD45RA^−^CD90^+^.

To conclude, we report the selective expression of CD93 on immature CML cells and the associated self-renewing nature of this primitive cell subpopulation. As CD93 is expressed on endothelial cells, platelets and other critical cell types, it is unlikely that CD93 can be targeted therapeutically [16, 17]. CD93 has been previously described as an immature marker on a rare CD34^−^ population of human HSCs, which have self-renewal and repopulating capacities [36], and on AML LSCs [18, 37], most specifically on MLL-rearranged AML. Therefore, it is likely that there is a role for CD93 within stem cell phenotypes, although its precise function(s) remains to be elucidated. In preliminary experiments, we are exploring whether CD93 had a role in LSC adherence within the BM niche. CD93 has been identified as a key mediator of integrin α5β1 interaction with fibronectin [38]. Furthermore, it has been previously reported that α5β1 is implicated in CP-CML LSC binding to fibronectin through CD44 [39, 40]. This will be fully evaluated in ongoing and future studies. Finally, identifying primitive CML cells with self-renewing capability, which persist throughout treatment, and may be responsible for molecular recurrence, is becoming increasingly important in an era where TKI withdrawal is becoming standard-of-care for some patients. The possible discovery of a predictive biomarker, namely CD93, to distinguish those patients at high risk of molecular recurrence, if validated, could become an important test for patients considering TFR, and could also have the potential to identify patients suitable for future TFR trials.

## Supplementary information

Supplemental material
