# Correction: CD93 is expressed on chronic myeloid leukemia stem cells and identifies a quiescent population which persists after tyrosine kinase inhibitor therapy

**DOI:** 10.1038/s41375-020-0721-4

**Published:** 2020-01-31

**Authors:** Ross Kinstrie, Gillian A. Horne, Heather Morrison, David Irvine, Chinmay Munje, Eduardo Gómez Castañeda, Hothri A. Moka, Karen Dunn, Jennifer E. Cassels, Narissa Parry, Cassie J. Clarke, Mary T. Scott, Richard E. Clark, Tessa L. Holyoake, Helen Wheadon, Mhairi Copland

**Affiliations:** 1 Paul O’Gorman Leukaemia Research Centre, Institute of Cancer Sciences, University of Glasgow, Glasgow, UK; 2 Molecular and Clinical Cancer Medicine, University of Liverpool, Liverpool, UK

**Keywords:** Cancer stem cells, Chronic myeloid leukaemia

**Correction to: Leukemia**

10.1038/s41375-019-0684-5

This article was originally published under NPG’s License to Publish, but has now been made available under a CC BY 4.0 license. The PDF and HTML versions of the paper have been modified accordingly.

