# Supplementary material for: CD93 is expressed on chronic myeloid leukemia stem cells and identifies a quiescent population which persists after tyrosine kinase inhibitor therapy
Source: Leukemia. 2020 Jan 2;34(6):1613–25. doi: 10.1038/s41375-019-0684-5 (PMC7272220; doi:10.1038/s41375-019-0684-5)
Supplement: Supplementary file 1 — Supplemental material [file 41375_2019_684_MOESM1_ESM.pdf]

Table S1: Patient sample characteristics

Identifier	Age	Gender	Subtype	Breakpoint	Pre-treatment
**CML110**	48	F	CP	P210	None
**CML111**	NK	NK	CP	P210	NK
**CML332**	62	F	CP	P210	None
**CML339**	34	F	CP	P210	None
**CML340**	55	M	CP	P210	None
**CML342**	54	M	CP	P210	None
**CML343**	46	M	CP	P210	None
**CML347**	34	F	CP	P210	Imatinib
**CML350**	NK	NK	CP	P210	NK
**CML351**	NK	NK	CP	P210	NK
**CML373**	48	M	CP	P210	None
**CML385**	51	M	CP	P210	None
**CML388**	61	F	CP	P210	None
**CML391**	33	F	CP	P210	Interferon
**CML393**	35	M	CP	P210	None
**CML395**	34	M	CP	P210	None
**CML399**	51	M	CP	P210	None
**CML407**	50	M	CP	P210	None
**CML456**	58	M	CP	P210	None
DESTINY samples – Bone marrow MNCs					
Identifier		Subtype	Breakpoint	Relapsed on TKI discontinuation	
**046-325**		CP	P210	Yes	
**069-305**		CP	P210	Yes	
**161-302**		CP	P210	Yes	
**364-064**		CP	P210	No	
**364-067**		CP	P210	No	
**364-078**		CP	P210	Yes	
**364-082**		CP	P210	No	
**364-084**		CP	P210	No	

NK = not known

Table S2: Primer sequences

	Forward	Reverse
ABL-A3-B	GTTTGGGCTTCACACCATTCC	-
ATM	CGGAGCTGATTGTAGCAACATACTA	CAGATAGAGCCTGAAGTACACAGAG
ATR	CAGCTCTCTATGAAGGCCATTCAA	GTTCTACTGTTTCACTGTCTGTTGC
BCL2	ATCGCCCTGTGGATGACTGAGT	GCCAGGAGAAATCAAACAGAGGC
BCR-C5R	BCR-C5R	-
BMI1	GCATCGAACAACGAGAATCA	GCTGGTCTCCAGGTAACGAA
C-JUN	CTTGAAAGCTCAGAACTCGGAG	CTGCGTTAGCATGAGTTGGC
CCND1	CAGAAGGAGATTGTGCCATCC	GAAGCGGTCCAGGTAGTTCA
CCNA1	CTCGTAGGAACAGCAGCTATGC	GCTAGAACTTTCAGAAGCAAGTGTTC
CCNA2	TGGCGGTACTGAAGTCCGG	CAAGGAGGAACGGTGACATGC
CCNB1	CAGCTCTTGGGGACATTGGTAAC	ACTGGCACCAGCATAGGTACC
CCND1	CAACGTGCAAGCCTCGGA	CAAAGTGCTGATCCCTTAAGTATGTC
CCND2	CAAGTTGATGCTCTTAAAGATGCTCC	GCAGCAGTCAGTATTCTGTACTGG
CD79A	GAACGAGAAGCTCGGGTTG	TGCCCACATCCTGGTAGGT
CD93	GGCAGACAGTTACTCCTGGGTT	GGAGTTCAAAGCTCTGAGGATGG
CDC25A	GTCTAGATTCTCCTGGGCCATTG	CAGAATGGCTCCTCTTCAGAGC
CDC25B	GGATTTGTGGACATCCTAGAGAGT	ACTTGCTGTACATGACGAGGT
CDC25C	CACTCAGCTTACCACTTCTGCAG	GGGCTACATTTCATTAGGTGCTGG
CDK2	GCCTGATTACAAGCCAAGTTTCC	TCCGCTTGTTAGGGTCGTAGT
CDK4	GAAACTCTGAAGCCGACCAG	AGGCAGAGATTCGCTTGTGT
CDK6	CCTGCAGGGAAAGAAAAGTGC	CCTCCTCTTCCCTCCTCGAA
CDKN2A	CCGAATAGTTACGGTCGGAGG	CACGGGTCGGGTGAGAGT
CDKN2B	GGTGGCTACGAATCTTCCG	CCTAAGTTGTGGGTTCACCA
CHEK1	CCATCAGCAAGAATTACCATTCCAG	CTGGGAGACTCTGACACACC
CHEK2	ATGAGAACCTTATGTGGAACCCC	GCTCAGAGAAAGGTGGATACCC
CSF1R	GGACATTCATCAACGGCTCT	GCTCAGGACCTCAGGGTATG
CSF2R	GTTACCACACCCAGCATTCC	TTGGCAGTCCCAGCTTAAAT
CSF3R	TGGGACCCAGGAATCTATCA	ATGAGGCAGGAGAGGTTGTG
CYC1	ACTGCGGGAAGGTCTCTACTT	GGGTGCCATCGTCAAACTCTA
CYCLIN D3	TGGATGCTGGAGGTATGTGA	TGCACAGTTTTTCGATGGTC
CYCLIN E1	GTCCTGGCTGAATGTATACATGC	CCCTATTTTGTTCAGACAACATGGC
DTX1	AATCCCGAGGATGTGGTTCG	GTAGCCTGATGCTGTGACCA
E2F1	GGACCTGGAAACTGACCATCAG	CAGTGAGGTCTCATAGCGTGAC
E2F2	CTCTCTGAGCTTCAAGCACCTG	CTTGACGGCAATCACTGTCTGC
E2F3	AGCGGTCATCAGTACCTCTCAG	TGGTGAGCAGACCAAGAGACGT
EFNA1	GTCTGAGAAGTTCCAGCGCT	CACTGACAGTCACCTTCAACC
EFNA4	GAGGCTCCAGGTGTCTGTCT	AATGCTCCATCTTGTCGGTCTG
EGR1	TGACCGCAGAGTCTTTTCCT	TGGGTTGGTCATGCTCACTA
EGR2	CTGACACTCCAGGTAGCGAG	GTTGATCATGCCATCTCCGGC
ENOX2	GAGCTGGAGGGAACCTGATTT	CACTGGCACTACCAAACTGCA
ETS1	GAGTCAACCCAGCCTATCCA	ATGGGATGGAGCGTCTGATA
FBXW7	CCTTCTCTGGAGAGAGAAATGC	CTGTCTGATGTATGCACTTTTCC

FOXM1	TCAAAACCGAACTCCCCCTG	GCAGCACTGATAAACAAAGAAAGA
FOXO1	CTACGAGTGGATGGTGAAGAGC	CCAGTTCCTTCATTCTGCACTCG
FOXO3	CCTACTTCAAGGATAAGGGCGAC	GCCTTCATTCTGAACGCGCATG
FURIN	CCACATGACTACTCCGCAGAT	TACGAGGGTGAACTTGGTCAG
GATA3	CGAACTGTCAGACCACCACA	GGTTTCTGGTCTGGATGCCTT
GEMIN2	TTGTTACCTGAGGCTCATTCAC	TCATCAGCTAAATCACGTTGGT
GFI1	GCTCCCCAGGACCAGACTAT	CTTCGGTCAGCTGCGATT
GSK3B	CGACTAACACCACTGGAAGCT	GGATGGTAGCCAGAGGTGGAT
HES1	GGAGAAAAATTCCTCGTCCC	CGCGAGCTATCTTTCTTCAG
HLF	GCCCATGATCAAGAAAGCTC	GGCGATCTGGTTCTCTTTCA
HOXA4	ATGTCAGCGCCGTTAACCC	TGTTGGGCAGTTTGTGGTCT
HOXA5	AAGTCATGACAACATAGGCGGC	GATACTCAGGGACGGAAGGC
HOXA6	TGCGCGGGTGCTGTGTAT	GCTGCGTGGAATTGATGAGC
HOXB4	CTGGATGCGCAAAGTTCACGTG	CGTGTCAGGTAGCGGTTGTAGT
ICAT	CATGCTGCGGAAGATGGGAT	GGAAAACGCCATCACCACGT
IKZF1	AACGTCGCCAAACGTAAGAG	AGTTGATGGCGTTGTTGATG
INHB1	AAGTCGGGGAGAACGGGTAT	GGTCACTGCCTTCCTTGGAA
INK4A	CTCGTGCTGATGCTACTGAGGA	GGTCGGCGCAGTTGGGCTCC
INK4C	CGTCAATGCACAAAATGGATTTGG	GAATGACAGCGAAACCAGTTCGG
INK4D	GTGCATCCCGACGCCCTCAAC	TGGCACCTTGCTTCAGCAGCTC
MDM2	TGTTTGGCGTGCCAAGCTTCTC	CACAGATGTACCTGAGTCCGATG
MEIS1	AAGCAGTTGGCACAAGACACGG	CTGCTCGGTTGGACTGGTCTAT
MFNG	CTGGTACAGTTCTGGTTTGC	ATGTGTCCATGAAACGGGAGC
MPL	ACTCAGCGAGTCCTCTTTGTGG	CATAGCGGAGTTCGTACCTCAG
MPO	GCATCATCGGTACCCAGTTC	GTGGTGATGCCTGTGTTGTC
MYC	GACTCTGAGGAGGAACAAGA	TTGGCAGCAGGATAGTCCTT
NFKB1	AGATGATCCATATTTGGGAAGGC	TTGCTCTAATATTTGAAGGTATGGGC
NOTCH 1	TCCACTGTGAGAACAACACGC	ACTCATTGACATCGTGCTGGC
P190 BCR-e1-A	GACTGCAGCTCCAATGAGAAC	-
P210 BCR-e1-A	GAAGTGTTTCAGAAGGCTTCTCC	-
p38	GAGCGTTACCAGAACCTGTCTC	AGTAACCGCAGTTCTCTGTAGGT
PBX1	GGAGGATACAGTGATGGACTCG	GGAGGTATCAGAGTGAACACTGC
PML	CCGTCATAGGAAGTGAGGTCTTC	GTTTTCGGCATCTGAGTCTTCCG
PREP1	ATTGCATCAGGAGTCGCACAGC	GAGACTGAAGGCTGTCCACGTT
PSEN1	GGTGGTTCTGTATAAATACAGGTGC	AACAGTAATGTAGTCCACAGCAA
RUNX1	CACCTACCACAGAGCCATCAA	CTCGGAAAAGGACAAGCTCC
SPI1	GTGCCCTATGACACGGATCT	AAGCTCTCGAACTCGCTGTG
STIL	GACACAGTGCAAGCTGGAAGAC	AGTCAGGCTCTTGATCCTCACC
TP53	TTCTTGCATTCTGGGACAGCC	GGGGGTGTGGAATCAACCC
TP73	ACTTTGAGATCCTGATGAAGC	GAGGACCGGCCCGTAGGA
TYW1	ATTGTCATCAAGACGCAGGGC	GTTGCGAATCCCTTCGCTGTT
UBE2D2	CCATGGCTCTGAAGAGAATCC	GATAGGGACTGTCATTTGGCC
VEGF	AGAAGGAGGAGGGCAGAATCA	AGGGTACTCCTGGAAGATGTCC
WWTR1	GAGGACTTCCTCAGCAATGTGG	CGTTTGTTCCTGGAAGACAGTCA

Figure S1: Cell surface markers are deregulated between normal and CP-CML HSCs

Mean expression levels between normal HSCs (n=3) and CP-CML (n=6) from GSE47297 were normalised across each gene (z score) and represented using heatmap.2 in R/Bioconductor. Yellow represents increased expression and blue represents decreased expression within the data set. Samples could be clustered into disease process, i.e. normal and CP-CML.

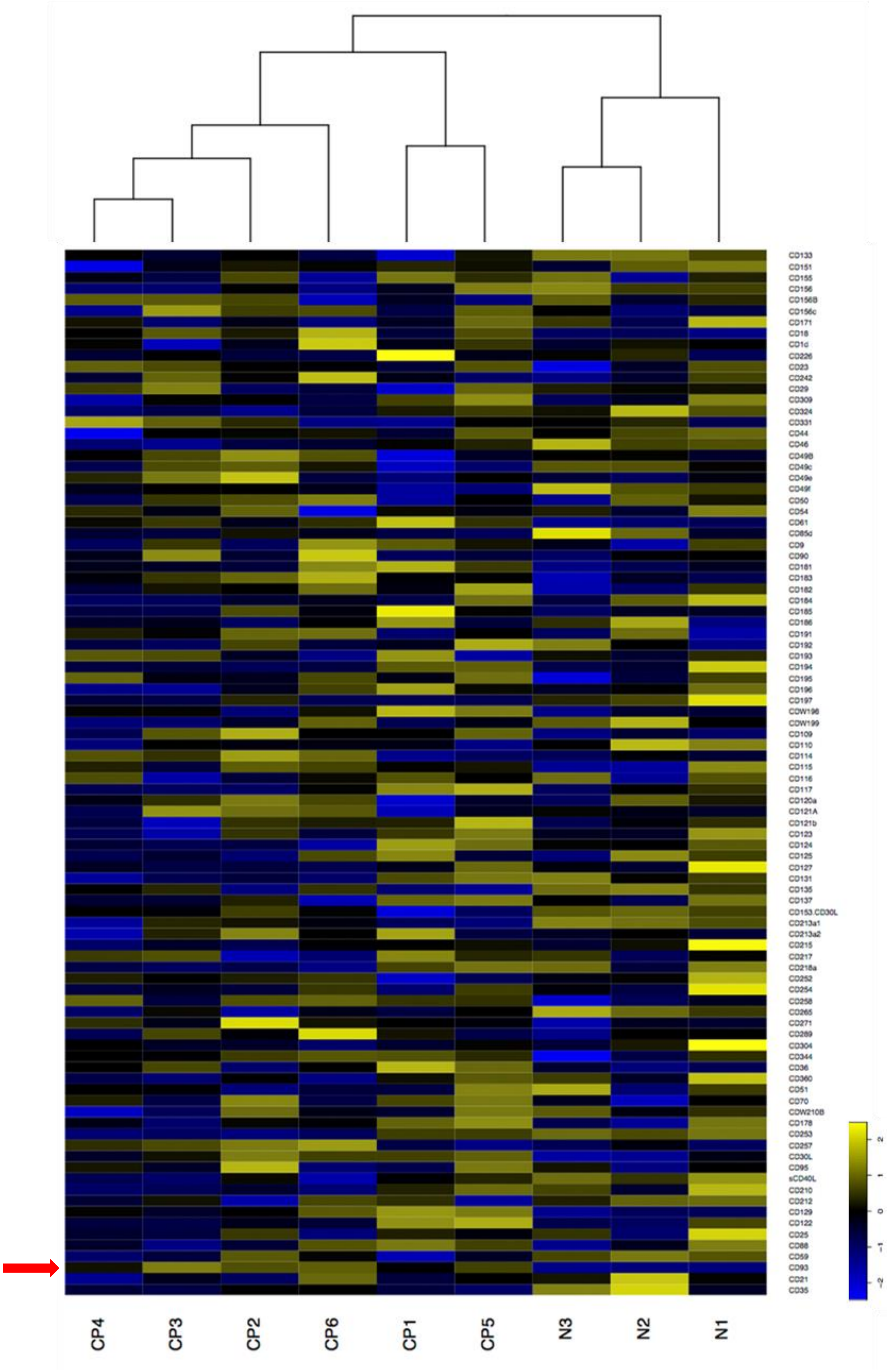

Figure S2: Sorting strategy for Lin$^-$CD34$^+$CD38$^-$CD90$^+$CD93$^{+/-}$ populations

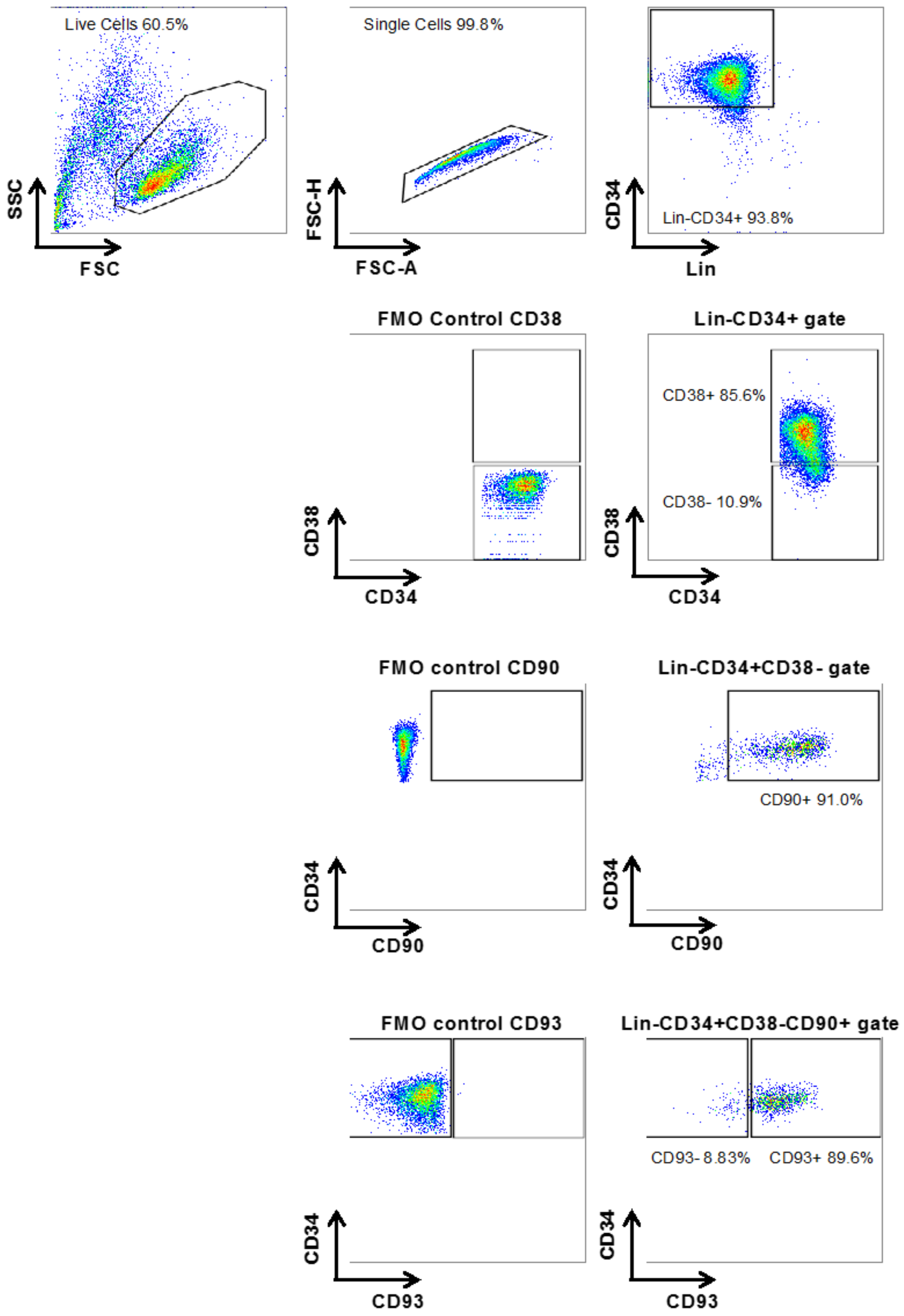

Figure S3: *BCR-ABL* analysis by FISH prior to NSG engraftment model

Chromosome t(9;22) *BCR-ABL* fusion was detected by a dial colour dual fusion probe (Abbott Molecular). Upon thawing, all samples were *BCR-ABL* positive, with only one sample (CML395 = CML5 in text) being less than 100%.

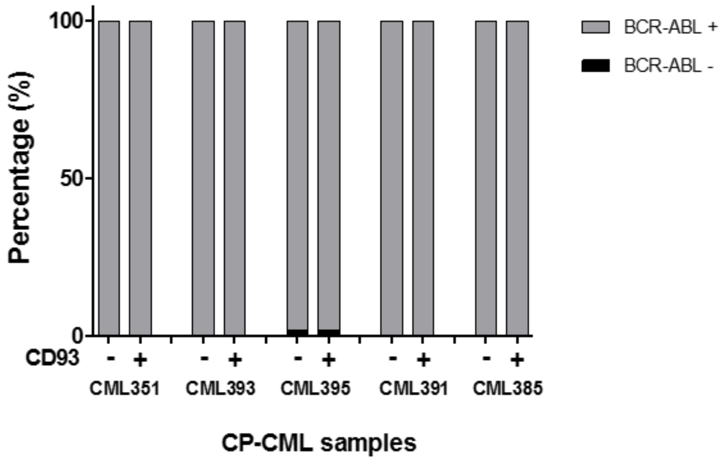

Figure S4: Engraftment was not identified at 8 weeks in $CD93^{+}$ or $CD93^{-}$ populations

Blood sampling was performed at 8 weeks. $CD45^{+}CD33^{+}$ cells were determined as a percentage of total cells. There was no statistical difference between the experimental arms at 8 weeks, suggesting that either engraftment had not yet occurred, or that engraftment potential was unable to be determined from peripheral blood sampling. n=3 CP-CML samples (CML1, CML2, and CML5 in text), p-value calculated from paired student's T test between arms of the experiment for each CP-CML sample.

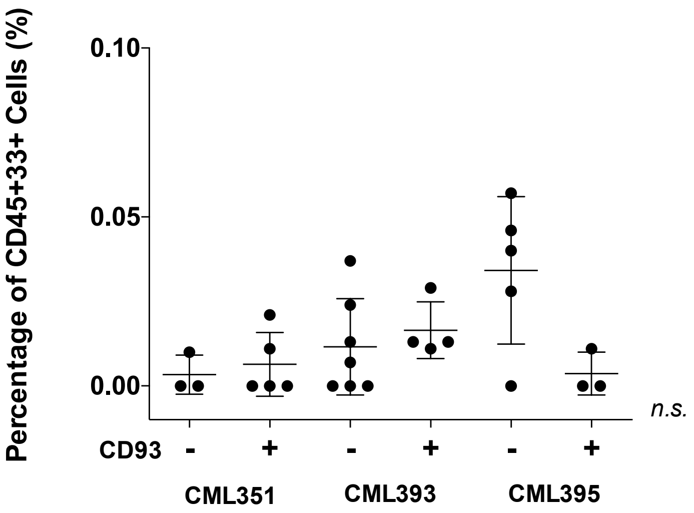

Figure S5: Non-*BCR-ABL* engraftment leads to multi-lineage cell potential in sample CML395 (CML5 in text)

(A) Human cell engraftment was characterized by percentage of $CD45^{+}CD33^{+}$. (B) Human $CD45^{+}$ cells were isolated by FACS sorting and analysed by FISH for the *BCR-ABL¬* gene rearrangement. Percentage of *BCR-ABL* positive cells, as determined from analysis of 100 cells, was assessed for each murine experiment. All mice within the $CD93^{-}$ experimental arm were predominantly *BCR-ABL* negative, with a variably sized BCR-ABL positive population evident within the $CD93^{+}$ arm. (C) Example of FACS analysis isolating both myeloid and lymphoid lineages within the $CD45^{+}$ sample.

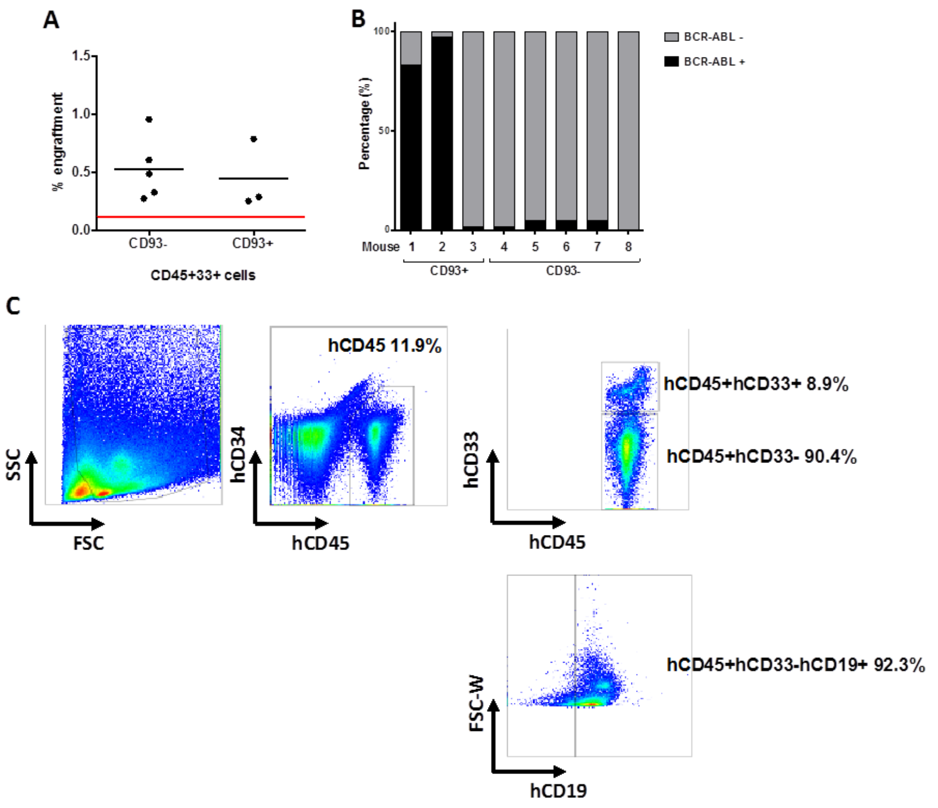

Figure S6: Frequency of gene expression within single cell analysis and STRING map of genes with increased frequency related to *GATA1*

A

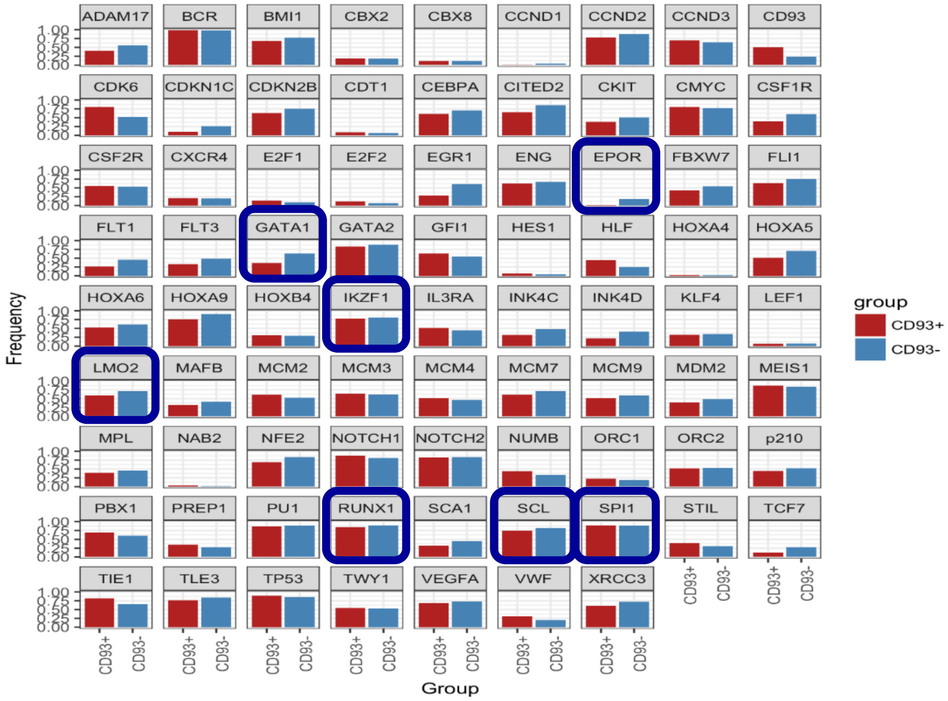

B

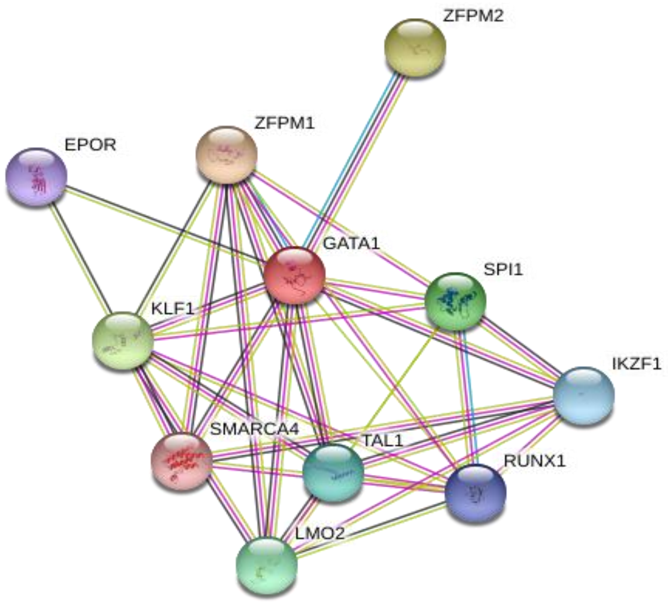

Figure S7: Gene clustering cannot clearly discriminate between CD93+ and CD93- populations at single cell level

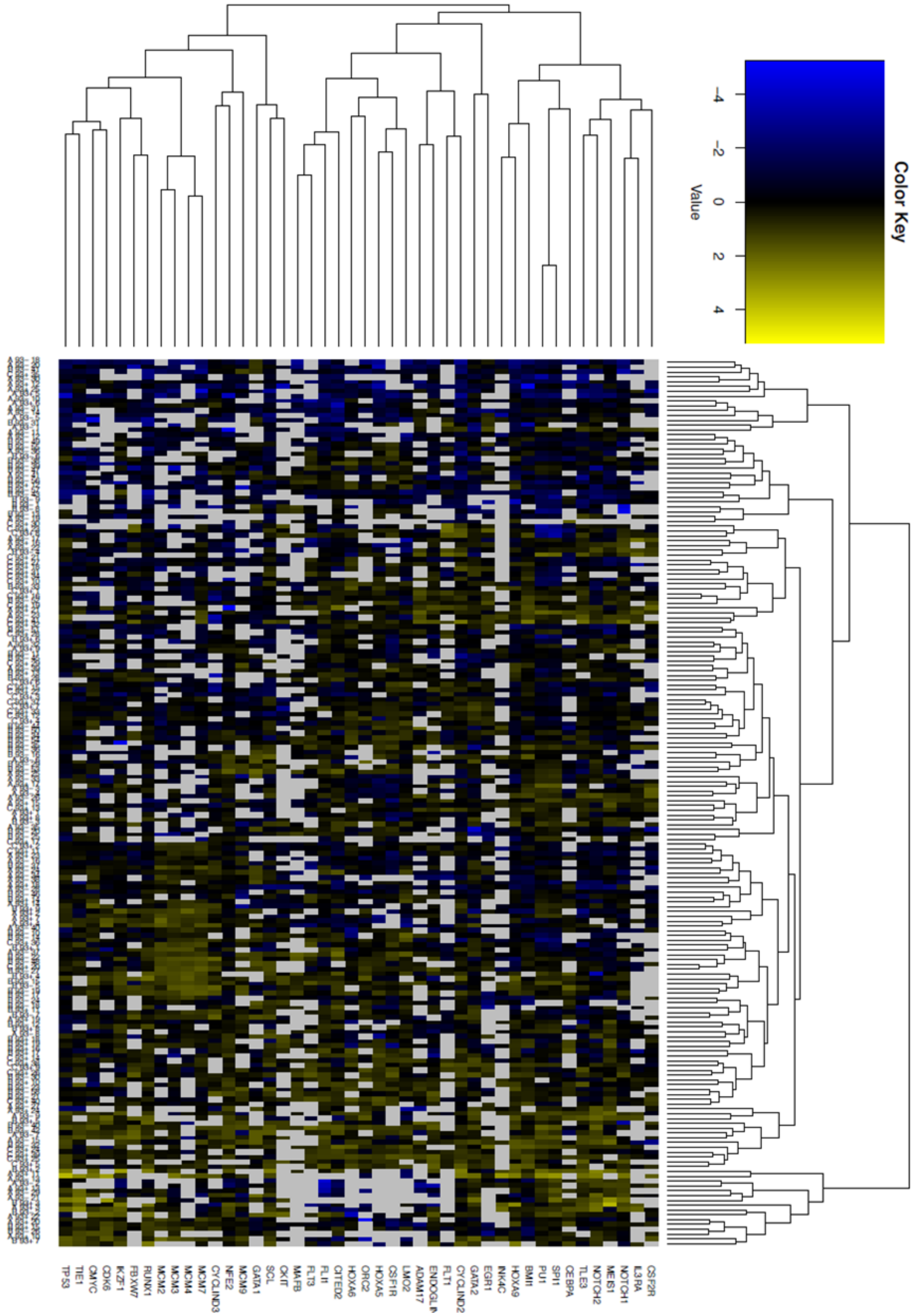

Figure S8: *BCR-ABL* expression in CD93-selected LSC populations

(A) To asses *BCR*-ABL expression, FISH for the *BCR-ABL* gene rearrangement was used. Percentage of *BCR-ABL* positive cells, as determined from analysis of a minimum of 100 cells, was assessed for each murine experiment where cell numbers allowed (n=2). (B) Representative FISH images of *BCR-ABL* expression in lin$^-$CD34$^+$CD38$^-$CD90$^+$CD93$^+$ and lin$^-$CD34$^+$CD38$^-$CD90$^+$CD93$^-$ populations.

A

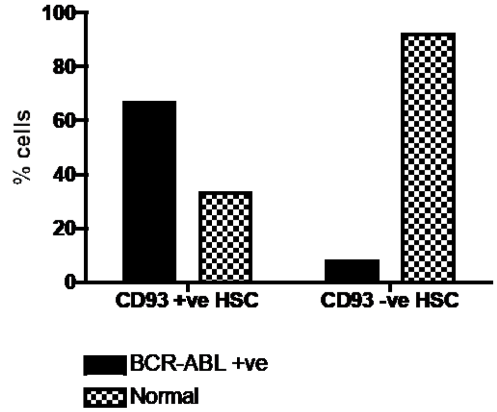

B

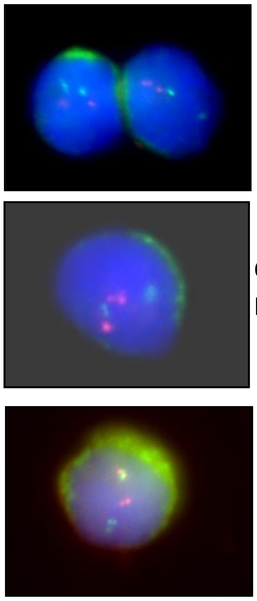

CD93- LSC
Normal R2G2

CD93+ LSC
Normal R2G2

CD93+ LSC
BCR-ABL+ R1G1F1
